# A False Pituitary Tumor

**DOI:** 10.1210/jcemcr/luad054

**Published:** 2023-06-01

**Authors:** Maria D Hurtado Andrade, Elif Tama, John L D Atkinson, Alice Y Chang

**Affiliations:** Division of Endocrinology, Diabetes, Metabolism, and Nutrition, Department of Internal Medicine, Mayo Clinic, Jacksonville, FL 32224, USA; Division of Endocrinology, Diabetes, Metabolism, and Nutrition, Department of Internal Medicine, Mayo Clinic, Jacksonville, FL 32224, USA; Department of Neurosurgery, Mayo Clinic Rochester, MN 55905, USA; Division of Endocrinology, Diabetes, Metabolism, and Nutrition, Department of Internal Medicine, Mayo Clinic, Jacksonville, FL 32224, USA

**Keywords:** pituitary tumor, intracranial hypotension, CSF leak

## Abstract

A false pituitary tumor describes pituitary enlargement due to intracranial hypotension. Reported previously primarily in the neurological literature, we present this case referred to endocrinology for evaluation of a pituitary mass. A 24-year-old male was referred to endocrinology for evaluation of pituitary enlargement without a hypo-enhancing lesion on magnetic resonance imaging (MRI). The main symptom reported was headache that was worse in the standing position and in the afternoon. He had no symptoms or signs of pituitary mass-effect, or hormone excess or deficiencies. Past medical history was relevant for a history of nerve schwannoma status post resection with subsequent spinal fusion. Biochemical evaluation of pituitary hormones was normal. Upon review of his pituitary MRI, other abnormalities seen were suggestive of intracranial hypotension. Based on his history and imaging findings, he was diagnosed with intracranial hypotension causing a “false pituitary tumor” rather than pituitary enlargement or abnormality. Further evaluation revealed multiple spinal leaks that were patched. His symptoms subsided within a few days of repair. Endocrinologists should be aware of the possible misdiagnosis of a pituitary mass due to intracranial hypotension.

## Introduction

Referrals for pituitary masses or enlargement are increasingly encountered in the endocrine clinical practice, mostly related to incidental findings due to increased imaging for other reasons [1]. Pituitary masses are the most common cause of sellar masses or enlargement, and account for up to 20% of all intracranial neoplasms [2]. Benign pituitary adenomas account for the majority (more than 90%) of pituitary masses or enlargement [3, 4]. Less common causes of pituitary masses or enlargement include pituitary hyperplasia (eg, pregnancy-related lactotroph hyperplasia; thyrotroph and gonadotroph hyperplasia due to prolonged uncontrolled/untreated primary hypothyroidism and hypogonadism, respectively), cysts, abscesses, inflammation (ie, hypophysitis), primary malignancy, and metastatic disease. Furthermore, parasellar masses can be mistaken for pituitary enlargement, such as meningiomas. Pituitary masses or enlargement can present with neurologic symptoms, including visual field deficit from optic nerve compromise and cranial nerve palsies from direct extension of the mass into the cavernous sinuses, signs and symptoms related to under- or oversecretion of pituitary hormones, or as an incidental finding on radiologic examination performed for another reason. Magnetic resonance imaging (MRI) is the single best imaging procedure for most sellar masses or sellar enlargement [5]. In gadolinium-enhanced images, pituitary adenomas, as well as other sellar masses, take up gadolinium at a lesser degree than the normal pituitary and therefore present as hypo-enhancing pituitary lesions. When patients are referred for pituitary abnormalities, a thorough clinical evaluation in conjunction with pituitary hormone testing and MRI can lead to the correct diagnosis and adequate treatment. Here, we present a rare case of a patient referred to us for evaluation of atypical orthostatic headaches and a pituitary tumor or mass who was diagnosed instead with a “false pituitary tumor” or appearance of pituitary enlargement secondary to intracranial hypotension.

## Case Presentation

A 24-year-old male was referred to our clinic for a pituitary tumor/mass discovered during evaluation of progressively worsening headaches over 5 years. He described them as a sensation of increased pressure or diffuse headache occurring in the afternoon, positional or orthostatic (worse while standing), particularly behind his eyes, precipitated by physical activity, and accompanied by photophobia, blurry vision, nausea, and projectile vomiting. Prior to presentation, evaluation for headaches at his local hospital included brain MRI that identified a pituitary tumor by report.

Past medical history included a history of C7 to T1 nerve schwannoma status post resection and subsequent spinal fusion from C6 to T1, 8 years prior to his presentation. One year later, due to instrumentation failure, he underwent fusion extension from C4 to T3 as well as wound revision for poor wound healing.

Review of systems was remarkable for intentional weight loss of 60 pounds in 1 year due to dietary changes and increased physical activity. He denied visual field deficits and did not have any symptoms or signs of pituitary hormone excess or deficiency. On confrontation, he had no evidence of visual field deficits.

## Diagnostic Assessment

**Figure 1.**
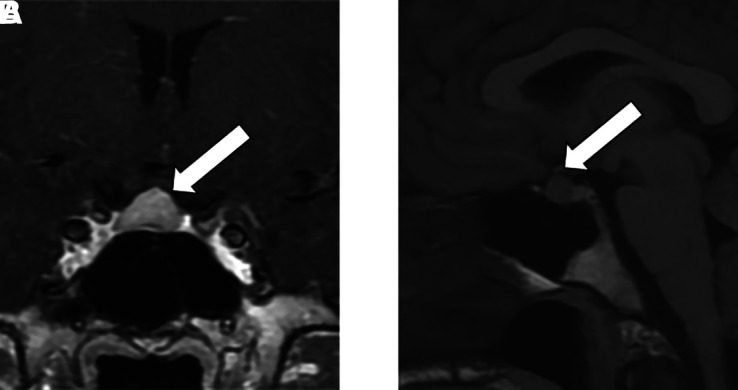
Coronal (A) and sagittal (B) views of the pituitary gland in a brain MRI. The pituitary is mildly enlarged with rounded convexity of the superior margin (white arrows).

A repeat MRI with and without intravenous contrast at our institution revealed a mildly enlarged and diffusely enhancing pituitary with rounded convexity of the superior margin (Fig. 1). There were no discrete hypo-enhancing lesions identified. The pituitary infundibulum deviated to the right but was otherwise normal appearing. Additional findings included: downward shift of the mammillary bodies, the pons, and cerebellar tonsils, shortening of the mid-sagittal width of the interpeduncular fossa cistern, and partially effaced inferior portion of the fourth ventricle and cerebral aqueduct. All these findings were suggestive of intracranial hypotension and were not present in an MRI from 8 years before.

Endocrine laboratory testing, including IGF-1, prolactin, FSH, LH, testosterone, TSH, free T4, ACTH, and cortisol levels, was normal.

Upon further evaluation by neurosurgery, the history of positional or orthostatic headaches and prior spine surgeries and imaging findings suggestive of intracranial hypotension, it was suspected that this young man had cerebrospinal fluid (CSF) volume depletion syndrome manifesting with orthostatic headaches. Upon review of prior images, he had evidence of extradural CSF accumulation in the spine suggestive of CSF leak. This finding had been present since his 1-year follow-up imaging after spinal fusion and persisted at his 2-year and 3-year follow-up imaging. He also had evidence of brain sagging as early as 1 year after his spinal fusion. In the setting of diffuse pituitary gland enhancement without hormonal abnormalities, it was concluded that the pituitary gland findings on MRI were not a pituitary tumor but rather a pituitary enlargement due to anatomical adaptation from CSF volume depletion.

## Treatment

Our patient underwent a dynamic computed tomography myelogram for leak localization and repair. The myelogram revealed CSF leaks at the T1-T2, T3, and T5 vertebral body levels. The study guided placement of multilevel blood patches at these sites. After the procedure, the patient's headaches completely resolved in 3 days.

## Outcome and Follow-up

Although his spinal CSF leaks were likely related to his schwannoma and his prior surgeries, his history of poor wound healing raised the possibility of connective tissue disorder that predisposes to spontaneous CSF leak. He was evaluated by the genetics department to rule out common connective tissue disorders such as Marfan and various types of Ehlers-Danlos syndromes. Genetic testing was completed and was negative for relevant connective tissue disorders. Our patient did not undergo follow-up imaging at our institution to assess resolution of intracranial anatomic changes related to intracranial hypotension.

## Discussion

**Figure 2.**
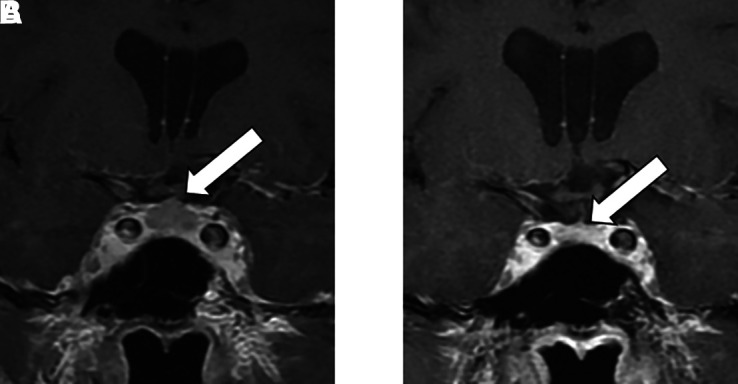
MRI imaging of the pituitary gland (white arrows) before (left) and 6 months after (right) a spinal blood patching in a patient with CSF leak (imaging corresponding to another patient with CSF leak resulting in enlargement of the pituitary gland; case and images courtesy of Dr. John L.D. Atkinson).

Endocrinologists should be aware of the presentation of false pituitary tumors with enhancing pituitary enlargement without a hypo-enhancing lesion compatible with a pituitary tumor or evidence of pituitary hormone secretion or deficiency. Clinically, patients with CSF volume depletion characteristically present with orthostatic headaches that usually develop in the afternoon [6]. CSF volume depletion is commonly associated with intracranial imaging abnormalities. Prominent abnormal features include diffuse meningeal enhancement, sagging of the brain with tonsillar herniation and descent of the brainstem, engorgement of cerebral venous sinuses, decrease in the size of cisterns and ventricles, increased anteroposterior diameter of the brainstem, flattening of the optic chiasm, and pituitary enlargement [7]. Pituitary enlargement is often but not always symmetric. In patients with enlarged pituitary gland, pituitary hormonal abnormalities are usually not present [8, 9]. Not all cases present with all these abnormalities. The prevailing etiology of CSF volume depletion is that of spinal CSF leak due to trauma, spinal instrumentation, or connective tissue abnormalities [6]. If CSF volume depletion is related to a leak, localization is important because symptoms and imaging abnormalities resolve after epidural blood patching and/or surgical repair [7]. Orthostatic headaches can improve within days and imaging abnormalities, including pituitary enlargement, within months. Our patient’s headaches resolved in 3 days. As our patient did not undergo follow-up imaging at our institution, Fig. 2 corresponds to imaging of another patient with CSF leak resulting in enlargement of the pituitary gland before and after CSF leak repair (case and images courtesy of Dr. John L.D. Atkinson).

Equally relevant to endocrinologists, it is important to note that opposite of what happens with intracranial hypotension, intracranial hypertension can present with empty sella. As a matter of fact, in patients with idiopathic intracranial hypertension, empty sella, partial or complete, is the most commonly reported MRI finding [10].— Empty sella is the results of flattening of the pituitary secondary to arachnoid mater and CSF herniation into the sella.

## Learning Points

- A “false pituitary tumor” should be considered in patients presenting with pituitary enlargement and other imaging findings of intracranial hypotension but without frank evidence of a pituitary hypo-enhancing lesion.
- Patients with intracranial hypotension usually present without pituitary laboratory abnormalities since they do not have a pituitary space-occupying lesion.
- The cardinal symptom of intracranial hypotension is orthostatic headache.
- Past medical and surgical histories should be reviewed if there is a high suspicion for CSF leak, as localization of the leak site should be a priority before treatment.
- Treatment for intracranial hypotension includes patching of the leaks after which symptoms and imaging abnormalities resolve.

## Contributors

All authors made individual contributions to authorship. M.D.H., A.C., and J.A. participated in the diagnosis and management of the case. M.D.H. and E.T. participated in preparing the manuscript. All authors reviewed and approved the final draft.

## Data Availability

All data presented about the case are available as per request.

## Abbreviations

CSF: cerebrospinal fluid
MRI: magnetic resonance imaging
